# Enhanced ethanol production and reduced glycerol formation in *fps1∆* mutants of *Saccharomyces cerevisiae* engineered for improved redox balancing

**DOI:** 10.1186/s13568-014-0086-z

**Published:** 2014-12-11

**Authors:** Clara Navarrete, Jens Nielsen, Verena Siewers

**Affiliations:** Department of Chemical and Biological Engineering, Chalmers University of Technology, Kemivägen 10, Göteborg, SE-41296 Sweden

**Keywords:** Saccharomyces cerevisiae, Ethanol production, Glycerol, Redox balancing

## Abstract

Ethanol is by volume the largest fermentation product. During ethanol production by *Saccharomyces cerevisiae* about 4-5% of the carbon source is lost to glycerol production. Different approaches have been proposed for improving the ethanol yield while reducing glycerol production. Here we studied the effect of reducing glycerol export/formation through deletion of the aquaglyceroporin gene *FPS1* together with expressing *gapN* encoding NADP^+^-dependent non-phosphorylating glyceraldehyde-3-phosphate dehydrogenase from *Streptococcus mutans* and overexpressing the ATP-NADH kinase gene *UTR1* from *S. cerevisiae*. This strategy will allow reducing the redox balance problem observed when the glycerol pathway is blocked, and hereby improve ethanol production. We found that our strategy enabled increasing the ethanol yield by 4.6% in the case of the best producing strain, compared to the reference strain, without any major effect on the specific growth rate.

**Electronic supplementary material:**

The online version of this article (doi:10.1186/s13568-014-0086-z) contains supplementary material, which is available to authorized users.

## Introduction

Ethanol is both in terms of market value and volume one of the most important products from the biotechnology industry. Even though this process is highly optimized there is still interest to improve the productivity, the robustness of the strains and the product yield (van Maris et al. [2006]; Hahn-Hagerdal et al. [2007]). There are different parameters that determine the economy of this industrial bioprocess; one of the most important ones is the price of the feedstock (Wyman and Hinman [1990]). Therefore, it is of utmost importance to increase the ethanol yield as well as the carbon source utilization. During ethanol production by *Saccharomyces cerevisiae*, glycerol is a major by-product, representing 4-5% of the carbon source consumption, in addition to biomass, carbon dioxide and a number of other by-products such as acetic acid, pyruvic acid or succinic acid (Nissen et al. [2000b]; Wyman and Hinman [1990]; Zhang and Chen [2008]; Oura [1977]).

**Figure 1:**
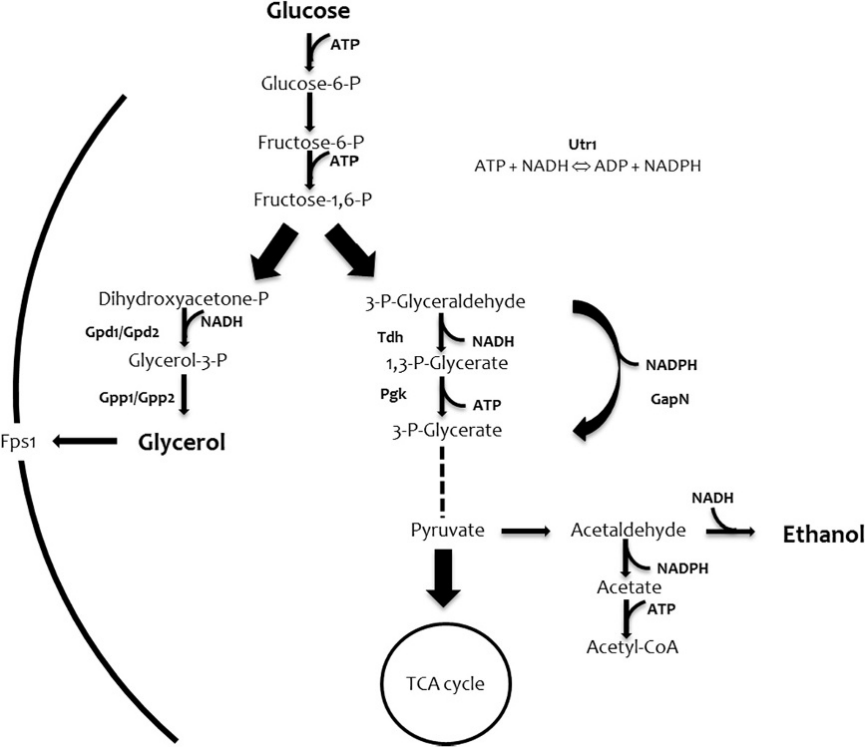
**Schematic diagram of ethanol and glycerol metabolism in** ***S. cerevisiae*** **.** Gpd1/Gpd2, glycerol 3-phosphate dehydrogenases; Gpp1/Gpp2, glycerol 3-phosphate phosphatases; Tdh, glyceraldehyde 3-phosphate dehydrogenase; Pgk, phosphoglycerate kinase; Fps1, glycerol exporter (protein channel); TCA, tricarboxylic acid cycle. The strategy used in this work consists of heterologous expression of *gapN* (encoding NADP^+^-dependent glyceraldehyde 3-phosphate dehydrogenase) from *S. mutans* and overexpression of *UTR1* (encoding ATP-NADH kinase) from *S. cerevisiae*.

During anaerobic fermentation, the respiratory chain is not functional and the NADH generated in connection with cell growth must be re-oxidized to NAD^+^ by formation of glycerol, in order to avoid an imbalance in the NAD^+^/NADH ratio (Nissen et al. [2000a]). Furthermore, under osmotic stress conditions, glycerol is produced and accumulated in the cell as an osmolyte, to protect cells against cell lysis (Andre et al. [1991]; Larsson et al. [1993]; Ansell et al. [1997]). Glycerol is synthethized from dihydroxyacetone phosphate in two steps catalysed by Gpd1/Gpd2 (glycerol-3-phosphate dehydrogenases) and Gpp1/Gpp2 (glycerol-3-phosphate phosphatases), respectively (Figure 1). Expression of *GPD1* and *GPP2* is induced by high osmolarity, whereas expression of *GPD2* and *GPP1* is stimulated under anaerobic conditions (Larsson et al. [1993]; Eriksson et al. [1995]; Nissen et al. [2000a]). It has been reported that the formation of glycerol could be decreased by the consumption of NADH by alternative metabolic pathways (Vemuri et al. [2007]; Bro et al. [2006]). It has also been shown that deletion of either the *GPD1* or *GPD2* gene led to a decrease in the glycerol yield (Guo et al. [2009]; Michnick et al. [1997]; Nissen et al. [2000a]), but the double *gpd1Δgpd2Δ* mutant had a dramatically reduced specific growth rate under aerobic conditions with growth being completely abolished at anaerobic conditions (Bjorkqvist et al. [1997]). To improve the ethanol yield while reducing glycerol formation, different approaches have been reported. To show whether a reduced formation of surplus NADH and an increased consumption of ATP in biosynthesis would result in a decreased glycerol yield and an increased ethanol yield in anaerobic cultivations, a yeast strain was constructed in which *GLN1* (glutamine synthetase) and *GLT1* (glutamate synthase) were overexpressed, and *GDH1* (NADP^+^-dependent glutamate dehydrogenase) was deleted (Nissen et al. [2000b]), which resulted in a 38% reduced glycerol yield. A genome-scale reconstructed metabolic network of *S. cerevisiae* was used to score the best strategies for metabolic engineering of the redox metabolism that would lead to decreased glycerol and increased ethanol yields, and this showed that expressing a non-phosphorylating, NADP^+^-dependent glyceraldehyde-3-phosphate dehydrogenase (GapN) was one of the best strategies tested (Bro et al. [2006]). This has been confirmed in several studies, and it has also been shown that expression of GapN can rescue the negative effects from deletion of the glycerol export system Fps1 (Bro et al. [2006]; Guo et al. [2009]; Wang et al. [2011]; Zhang et al. [2011]). GapN catalyses the irreversible conversion of glyceraldehyde-3-phosphate and NADP^+^ into 3-phosphoglycerate and NADPH in glycolysis (Figure 1). With this strategy, production of glycerol is substituted with production of ethanol involving a net oxidation of NADH (Bro et al. [2006]; Arnon et al. [1954]). In another strategy to reduce glycerol production the *Escherichia coli mhpF* gene, encoding an acetylating NAD-dependent acetaldehyde dehydrogenase, was expressed in a *gpd1Δ gpd2Δ* strain, and it was shown that anaerobic growth could be restored by supplementation with 2 g/l acetic acid accompanied by reduced glycerol production (Guadalupe Medina et al. [2010]).

Although it has been also shown that glycerol can cross the plasma membrane through a H^+^ symport, detected in cells grown on non-fermentable carbon sources, and by passive diffusion (Gancedo et al. [1968]; Lages and Lucas [1997]; Lages et al. [1999]; Oliveira et al. [2003]), glycerol is mainly exported across the plasma membrane through the protein channel Fps1 (Remize et al. [2001]) regulated by extracellular osmolarity (Tamas et al. [1999]). Fps1 is a member of the major intrinsic protein (MIP) family of channel proteins. MIP channels have been reported to contain six putative transmembrane domains (Walz et al. [1997]; Li et al. [1997]; Stamer et al. [1996]). Although Fps1 can transport glycerol in both directions, the main role of this glycerol facilitator is to regulate glycerol export rather than its uptake (Luyten et al. [1995]; Tamas et al. [1999]). The stress-activated protein kinase Hog1 can phosphorylate Fps1 triggering its endocytosis and further degradation under different stress conditions such as high acetic acid levels (Mollapour and Piper [2007]). Fps1 is required for glycerol export under anaerobic conditions, and mutants lacking this protein grow poorly under anaerobiosis (Tamas et al. [1999]). In an *fps1Δ* mutant, intracellular glycerol accumulates since it cannot be exported. As a result, the accumulation of glycerol inside the yeast cells may induce other regulatory systems in order to reduce glycerol biosynthesis, thus resulting in an increase of the ethanol yield (Zhang et al. [2007]).

NAD(H) kinases catalyse NAD(H) phosphorylation by using ATP or inorganic polyphosphate, constituting the last step of the NADP(H) biosynthetic pathway (Kawai et al. [2001]; McGuinness and Butler [1985]). *S. cerevisiae* is endowed with three NAD(H) kinase homologues, namely Utr1, Pos5 and Yel041w (Kawai et al. [2001]; Outten and Culotta [2003]; Strand et al. [2003]). The ATP-NAD kinase Utr1 was proposed to participate in the ferrireductase system by supplying NADP (Kawai et al. [2001]; Lesuisse et al. [1996]). Pos5 was described as an ATP-NADH kinase, located in the mitochondrial matrix and reported to play an important role in several mitochondrial processes requiring NADPH. Cells lacking Pos5 accumulate mutations in the mitochondrial DNA and show poor growth in the presence of glycerol, oxidative damage and when growing in medium without arginine (Strand et al. [2003]; Outten and Culotta [2003]). Later on, Yel041w (re-named as Yef1) and Utr1 were identified as ATP-NADH kinases (Shi et al. [2005]). Analysis of the single, double and triple mutants, which were found viable, showed the important contribution of Pos5 to the mitochondrial function and survival at high temperature (37°C). The contribution of Utr1 to growth in low iron medium was also reported to be critical (Shi et al. [2005]).

Here, we explored the combinatorial effect of reducing glycerol export and formation through deletion of *FPS1*, while expressing *gapN* from *S. mutans* and overexpressing the ATP-NADH kinase gene *UTR1* from *S. cerevisiae*. With this strategy we aimed to solve the resulting redox balance problem and, at the same time, to increase the ethanol yield. We also analysed the effect on glycerol reduction/ethanol production, by using a set of different plasmids for varying expression levels of *gapN* and *UTR1*.

## Materials and methods

### Strain construction and media

The *S. cerevisiae* strains and plasmids used in this study are described in Table 1. CN1, which was used as the reference strain and CN6, where *FPS1* was knocked-out, were transformed with empty plasmids. The *FPS1* gene was deleted in CN2 to CN6 strains. CN2 to CN5 were transformed with different combinations of plasmids carrying *gapN* and *UTR1* under control of the *TEF1* promoter, and expressing the mentioned genes in high or low copy number (Table 1). Agar plates of synthetic medium (6.7 g/l yeast nitrogen base w/o aminoacids, 0.75 g/l complete supplement mixture w/o uracil, 2% glucose, 2% agar, pH 6) or YPD (2% glucose, 2% peptone, 1% yeast extract, 2% agar) supplemented with 200 mg/l of G418, were used for selection of the strains. For the fermentations, yeast cells were grown in minimal synthetic medium (Verduyn et al. [1992]) containing 20 g/l glucose. The pH was adjusted to 5.2 with 2 M KOH before sterilization. In bioreactors, 10 mg/l of ergosterol and 420 mg/l of Tween80 were added to the medium for the anaerobic growth of *S. cerevisiae*.

**Table 1:** ***S. cerevisiae*** **strains and plasmids used in this work**

Strains	Genotype description	Reference
CEN.PK 113-11C	*MAT* a *his3Δ* *1 ura3-52 MAL2-8*^*c*^*SUC2*	(van Dijken et al. [2000])
CEN.PK 113-11C; p426; p423 (CN1)	*MAT* a *his3Δ* *1 ura3-52 MAL2-8*^*c*^*SUC2* p426TEF1 p423TEF1	This work
*fps1*∆; p426-GapN; p423-UTR1 (CN2)	*MAT* a *his3Δ* *1 ura3-52 MAL2-8*^*c*^*SUC2 fps1*∆ p426TEF1-GapN p423TEF1-UTR1	This work
*fps1*∆; p426-GapN; p413-UTR1 (CN3)	*MAT* a *his3Δ* *1 ura3-52 MAL2-8*^*c*^*SUC2 fps1*∆ p426TEF1-GapN p413TEF1-UTR1	This work
*fps1*∆; p416-GapN; p423-UTR1 (CN4)	*MAT* a *his3Δ* *1 ura3-52 MAL2-8*^*c*^*SUC2 fps1*∆ p416TEF1-GapN p423TEF1-UTR1	This work
*fps1*∆; p416-GapN; p413-UTR1 (CN5)	*MAT* a *his3Δ* *1 ura3-52 MAL2-8*^*c*^*SUC2 fps1*∆ p416TEF1-GapN p413TEF1-UTR1	This work
*fps1*∆; p426; p423 (CN6)	*MAT* a *his3Δ* *1 ura3-52 MAL2-8*^*c*^*SUC2 fps1*∆ p426TEF1 p423TEF1	This work
**Plasmids**		
pCIChE-KK004-Gap	*neo*^r^, integrative plasmid	(Kocharin [2013])
p426TEF1	*URA3*, 2 μ plasmid, *TEF1* promoter, *CYC1* terminator	(Mumberg et al. [1995])
p416TEF1	*URA3*, centromeric plasmid, *TEF1* promoter, *CYC1* terminator	(Mumberg et al. [1995])
p423TEF1	*HIS3*, 2 μ plasmid, *TEF1* promoter, *CYC1* terminator	(Mumberg et al. [1995])
p413TEF1	*HIS3*, centromeric plasmid, *TEF1* promoter, *CYC1* terminator	(Mumberg et al. [1995])
p426TEF1-GapN	*URA3*, 2 μ plasmid, *gapN* from *S. mutans*	This work
p416TEF1-GapN	*URA3*, centromeric plasmid, *gapN* from *S. mutans*	This work
p423TEF1-UTR1	*HIS3*, 2 μ plasmid, *UTR1*	This work
p413TEF1-UTR1	*HIS3*, centromeric plasmid, *UTR1*	This work

### Deletion of *FPS1*

The *FPS1* gene was replaced, by using the *loxP-kanMX-loxP* cassette obtained by PCR from the plasmid pUG6 (Wach et al. [1994]) and a bipartite strategy (Erdeniz et al. [1997]). Primers containing upstream and downstream homologous regions of *FPS1* were used for that purpose (Table 2). The PCR products were used for transformation of CEN.PK 113-11C, and the transformed cells were selected in G418-YPD medium. The selectable marker was removed afterwards by expression of the Cre recombinase from plasmid pSH47 under the control of a galactose inducible promoter (Guldener et al. [1996]). The correct integration of the fragment disrupting the coding sequence was further tested by PCR and sequencing.

**Table 2:** **Oligonucleotide primers used in this study**

Primer name	Primer sequence (5′-3′)	Restriction sites^*^
kanMX_FPS1_F	**TATTTTCGATCAGATCTCATAGTGAGAAGGCGCAATT** CAGTAGTTGGCATCAGAGCAGATTGTACTGAGAG	
kanMX_R	AAACTCACCGAGGCAGTTCCATAG	
kanMX_F	ATGGTCAGACTAAACTGGCTGACG	
kanMX_FPS1_R	**CATCCATGCGATACATCATGTATAGTAGGTGACCAGGCTGAGTTC** TACCGCCTTTGAGTGAGCTGATAC	
UTR1_F	CG*GGATCC* CGAAAACAATGAAGGAGAATGACATGAATA	*Bam* HI
UTR1_R	CG*GAATTC* CGGTAACATTATACTGAAAACCTTGCTTGA	*Eco* RI
GapN_F	CG*GGATCC* CGAAAACAATGACAAAACAATACAAAAACT	*Bam* HI
GapN_R	CC*ATCGAT* GGAGATCTTCACTTTATGTCAAAGACAACA	*Cla* I

Primer overhangs containing part of *FPS1* up/down sequences are depicted in bold letters.

^*^Restriction sites with corresponding restriction enzyme are represented in italics.

### Heterologous expression of *gapN* and overexpression of *UTR1*

The sequence of the NADP^+^-dependent glyceraldehyde 3-phosphate dehydrogenase gene *gapN* from *S. mutans* (Gene ID: 1028095) was obtained from plasmid pCIChE-KK004-Gap and cloned into p426TEF1 and p416TEF1, respectively, by using PCR and restriction enzymes (REs). *Bam* HI and *Cla* I sites were additionally included in the designed PCR-primers for that purpose (Table 2). The *UTR1* sequence (Gene ID: 853508) was obtained by PCR from CEN.PK 113-11C genomic DNA and cloned into p423TEF1 and p413TEF1 plasmids by REs. In this case, the *Bam* HI/*Eco* RI combination was used (Table 2). The constructs were checked by PCR for *gapN* or *UTR1* genes, digestion with the proper RE combination and sequencing.

### Batch cultivations in flasks and analytical methods

Seed cultures of yeast cells were grown during 20–24 hours in shake flasks containing 25 ml of minimal medium at 30°C and constant shaking (120 rpm). Micro-aerobic conditions were obtained by using 150 ml flasks covered with fermentation bungs and containing 100 ml of medium (Arnon et al. [1954]). The shake flasks were inoculated with an initial OD_600_ of 0.05 and grown at 30°C (120–140 rpm). Samples of 2 ml of culture were taken at the selected time-points for optical density and HPLC measurements. Supernatants centrifuged for 5 min at high speed (1 ml) were loaded to a HPX-87G column (Biorad, Hercules, CA) on a Dionex Ultimate 3000 HPLC (Dionex Softron GmbH, Jülich, Germany) to measure the concentrations of glucose, ethanol, glycerol and acetate in the cultures at the different time points. The samples were run at a flow rate of 0.6 ml/min at 65°C, using 5 mM H_2_SO_4_ as mobile phase. Fermentation experiments were repeated three independent times.

### Fermentation analysis in bioreactors

Yeast cell seed cultures were grown in 25 ml of minimal medium supplemented with 20 g/l of glucose, and incubated at 30°C for 22–24 hours (130 rpm). The experiment was performed in 1 l stirrer-pro vessels (DasGip GmbH, Jülich, Germany) with a working volume of 0.5 l, and bioreactors were inoculated at initial OD_600_ of 0.02. The cells were centrifuged and washed with 5 ml of water before inoculation.

The temperature was controlled at 30°C using a DasGip Bioblock integrated heating and cooling thermo well. Agitation was maintained at 600 rpm using an overhead drive stirrer with one Rushton impeller. The bioreactors were flushed with N_2_ at 1 vvm when anaerobic growth conditions were required. The pH was maintained constant at 5.0 by the automatic addition of 2 M KOH.

## Results

### Yeast cells expressing the *gapN*/*UTR1* genes show an increased ethanol production capacity and reduced glycerol production.

**Figure 2:**
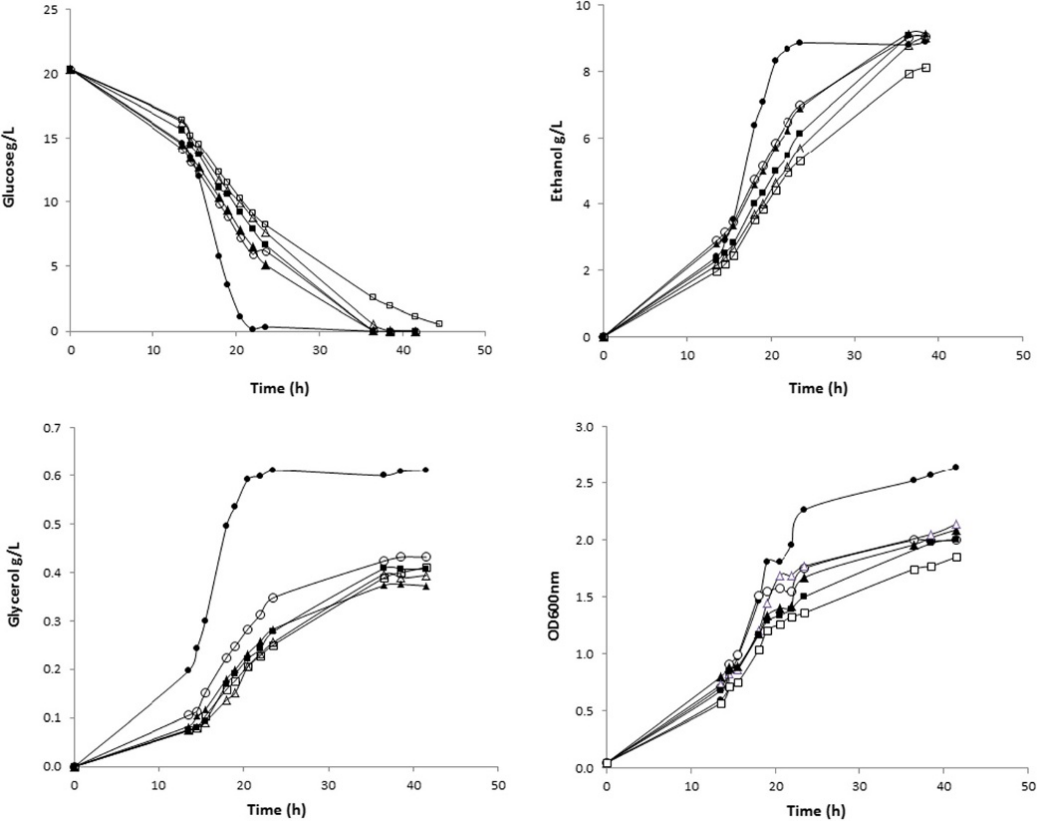
**Growth curves, glucose, ethanol and glycerol profiles during micro-aerobic conditions of** ***Saccharomyces cerevisiae*** **.** (●) CN1, (○) CN2, (▲) CN3, (△) CN4, (■) CN5, (□) CN6. The data are representatives derived from one of the three experiments performed.

Strains deleted in *FPS1* and expressing *gapN* and *UTR1* from either high or low copy number plasmids were cultivated in shake flasks under micro-aerobic conditions. Glucose consumption was completed in CN1 (reference strain) after around 30 h of fermentation. For the rest of strains, this level was reached first after about 37 h. The CN6 strain (*FPS1* deletion strain carrying both empty plasmids) showed 0.5-1 g/l of remaining glucose still after 45 hours of fermentation and the final ethanol production, for this strain, was much lower in comparison to the rest of the transformants tested (Figure 2). This correlates with the specific growth rate of the analysed strains (Table 3).

**Table 3:** **Compound yields, growth and specific ethanol productivity in engineered yeast strains**

Strain	Ethanol yield (g/g)^*^	Ethanol increase (%)	Glycerol yield (g/g)^*^	Acetate yield (g/g)^*^	μ (h^−1^)	q_sp_(g g^−1^ h^−1^)
CEN.PK 113-11C; p426; p423 (CN1)	0.449 ± 0.011	-	0.042 ± 0.018	0.0033 ± 0.00028	0.18 ± 0.048	26.55 ± 0.293
*fps1∆*; p426-GapN; p423-UTR1 (CN2)	0.466 ± 0.013	3.78	0.030 ± 0.012	0.0038 ± 0.00014	0.11 ± 0.059	51.71 ± 0.440
*fps1∆*; p426-GapN; p413-UTR1 (CN3)	0.470 ± 0.018	4.67	0.026 ± 0.010	0.0039 ± 0.00085	0.11 ± 0.052	52.02 ± 0.648
*fps1∆*; p416-GapN; p423-UTR1 (CN4)	0.457 ± 0.014	1.78	0.027 ± 0.012	0.0046 ± 0.00134	0.13 ± 0.046	40.47 ± 0.609
*fps1∆*; p416-GapN; p413-UTR1 (CN5)	0.458 ± 0.012	2	0.029 ± 0.013	0.0032 ± 0.00021	0.11 ± 0.038	52.37 ± 1.208
*fps1∆*; p426; p423 (CN6)	0.443 ± 0.0040	−1.33	0.023 ± 0.00049	0.0096 ± 0.00021	0.070 ± 0.0011	-

Data represent the means of results from triplicates ± SD.

*g/g is equivalent to gram of produced compound per gram of glucose.

μ represents the specific growth rate.

q_sp_ represents the specific ethanol productivity.

In the case of the reference strain, the highest level of ethanol was reached already after 25 h of fermentation, whereas for the rest of the strains we could observe a more gradual production over time until reaching the highest ethanol production (Figure 2). Ethanol production after 35–40 h of fermentation was improved in strains CN2 to CN5 expressing the alternative enzymes to reduce NADH levels (Figure 2). These results were confirmed when the specific ethanol productivity was calculated for these strains (Table 3). Strain CN6 grew very poorly hence affecting the calculation of ethanol productivity, and data for this strain were therefore not included in the further analysis.

Glycerol production was reduced in strains CN2 to CN6, confirming our initial hypothesis, showing only a minor effect on cell growth. An exception is strain CN6, which suffers from the lack of an NADH sink and hence has a much reduced growth rate (Figure 2 and Table 3).

Fermentation analysis in bioreactors showed that, except for the reference strain (CN1), cells were not able to grow under strict anaerobic conditions. Adaptation was necessary in order to complete the fermentation profile of the strains. This problem was partially solved by sparging air containing 12% oxygen at the beginning of fermentation. Under these conditions cells were able to grow, consume the carbon source and produce ethanol.

The ATP consumption by Utr1 and the inhibition of ATP formation by GapN, could be one of the reasons why the engineered strains showed a reduction in their ability to grow anaerobically.

Analytical methods revealed that results from individual experiments were similar to those obtained in shake flasks, with a higher ethanol production and glycerol reduction in the engineered strains (CN2-CN5) compared to the reference strain. But as a consequence of the previous adaptation needed, results were not very consistent between experiments and cells did not always grow at the same level, making it very difficult to represent the data as average yields of the different fermentation experiments. We concluded that more investigations are needed when growing cells in bioreactors, in order to use this strategy for industrial purposes, and therefore, we focused on the results we obtained in micro-aerobic conditions to analyse our strategy.

### Physiological characterization of the engineered yeast strains. Effect of the gene copy number of *gapN* and *UTR1* genes on ethanol yield

At the end of fermentation process, the ethanol yield was improved in the transformant strains by 1.7-4.6% (depending on the strain analysed) compared to the reference strain (CN1). CN2 and CN3 were the best ethanol producers (p-value < 0.05 after Student’s t-test), showing ethanol yields up to 0.47 grams per gram of glucose (Table 3). After the analysis of the possible effect of high/low expression of *gapN* and *UTR1*, by using different expression plasmids, we could observe the improvement in CN2 and CN3 strains both carrying *gapN* on a high-copy plasmid. In the case of *UTR1*, the copy number of the kinase gene did not seem to be crucial for a better ethanol yield, since the best producer (CN3) expressed *UTR1* from a low-copy plasmid. These results suggest a more important role of the NADP^+^-dependent glyceraldehyde 3-phosphate dehydrogenase in the redox balancing for improved ethanol production.

To further check this hypothesis, we performed an analysis of the *fps1Δ* strain expressing *gapN* or *UTR1* individually (both from a high-copy plasmid). The *fps1Δ* strain expressing *gapN* showed similar growth and final ethanol production compared to CN2 and CN3 strains, (7.98 g/l versus 8.14 and 8.43 g/l respectively) after ~40 h of fermentation in 20 g/l of glucose. On the other hand, the *fps1Δ* strain expressing *UTR1* grew similar to the CN6 control strain. Ethanol production at the end of fermentation was also at the same level as measured in the CN6 strain (5.6 g/l versus 6.7 g/l).

Furthermore, for the transformant strains the glycerol yield was successfully reduced by 25-40% compared to the reference strain. Although the transformant strains CN2-CN5 showed a specific growth rate reduced by 27-38%, they could still grow at a good level and entirely consume the carbon source. As explained before, the CN6 strain with highest glycerol reduction was the one most affected in terms of specific growth rate and also regarding ethanol production (Table 3 and Figure 2).

## Discussion

In *S. cerevisiae*, it is well known from previously published studies that, under aerobic conditions, *fps1Δ* mutants show a growth profile similar to a wild type, due to NADH re-oxidation by the respiratory chain (Zhang et al. [2007]). Under anaerobic conditions, glycerol production is the main redox-sink for the excess of NADH (Valadi et al. [2004]). When glycerol export is blocked, the *fps1Δ* mutants produce less glycerol and biomass, and show a lower glucose uptake rate compared to the reference strain. However, in case of the mutant strain, there is residual glucose in the growth medium, resulting in a decrease of total ethanol produced (Wang et al. [2011]). As already described in the results, the final ethanol production for the CN6 strain was much lower in comparison to the rest of the transformants tested (Figure 2). Moreover, it has been previously described that acetic acid enters glucose-repressed yeast cells primarily by facilitated diffusion through Fps1. The total loss of the channel also creates an acetate resistant phenotype by elimination of the major source of the acetic acid flux into the cell (Mollapour and Piper [2007]). The increased acetate yield observed in the CN6 strain might be connected to an inhibited acetate import in these cells (Table 3).

GapN catalyses the irreversible conversion of glyceraldehyde-3-phosphate and NADP^+^ into 3-phosphoglycerate and NADPH in glycolysis, and in contrast to the native yeast pathway, no NADH and ATP are released in this reaction (Figure 1). Expression of *gapN* has a high potential to reduce NADH generation, and may therefore lead to a complete redirection of flux from formation of glycerol to ethanol (Bro et al. [2006]). When alternative routes for NADH re-oxidation were expressed, ethanol production was improved in strains CN2 to CN5 (Figure 2).

The intracellular concentration of the NADP^+^/NADPH pool is much lower than that of the NAD^+^/NADH pool, and shifting part of the glycolytic flux from using NAD^+^ to using NADP^+^ as co-factor may therefore result in limitation of supply of this co-factor. We therefore evaluated whether increasing the NADP^+^/NADPH pool by over-expressing the *UTR1* gene encoding an NADH-kinase may have a positive effect on top of the use of GapN as a glycolytic enzyme. As already mentioned and represented in Figure 1, NAD(H) kinases catalyse NAD(H) phosphorylation by using ATP or inorganic polyphosphate in yeast, constituting the last step of the NADP biosynthetic pathway (McGuinness and Butler [1985]; Kawai et al. [2001]). *UTR1* overexpression does, however, not seem to lead to an effect on ethanol and glycerol production.

Regarding glycerol production, it is believed that the complete elimination of its production is not practical due to its importance in osmoregulation and maintenance of the intracellular redox balance (Blomberg and Adler [1992]; van Dijken and Scheffers [1986]). Moreover, it is a precursor used to synthesize the cellular membrane. On the other hand, it can be reduced to a minimal level in order to improve the ethanol yield (Bjorkqvist et al. [1997]; Nissen et al. [2000b]). Our hypothesis was confirmed and glycerol production was reduced in the strains CN2-CN6, Except for strain CN6, which suffers from the lack of an NADH sink, the cell growth was only mildly affected (Figure 2 and Table 3).

The present work evidences that the function of glycerol as a redox sink for anaerobic growth can be successfully replaced by introducing a heterologous GapN pathway from *S. mutans* in addition to deletion of the *FPS1* gene (responsible for glycerol export) and overexpression of the NADH-kinase Utr1, resulting in a reduction of glycerol (by-product) formation together with an increase in the ethanol yield.

By expressing *gapN* (in combination with the overexpression of *UTR1*) we can solve the redox problem in the *fps1Δ* mutant thereby increasing the specific growth rate and reducing the formation of NADH. Thus, the carbon source was redirected towards ethanol production in the engineered strains, which increased the ethanol yield by up to 4.6% compared to the reference strain.

## Authors’ original submitted files for images

Below are the links to the authors’ original submitted files for images. Authors’ original file for figure 1 Authors’ original file for figure 2
