# Supplementary figures and images for: Enhanced ethanol production and reduced glycerol formation in fps1∆ mutants of Saccharomyces cerevisiae engineered for improved redox balancing
Source: AMB Express. 2014 Dec 11;4:86. doi: 10.1186/s13568-014-0086-z (PMC4883998; doi:10.1186/s13568-014-0086-z)

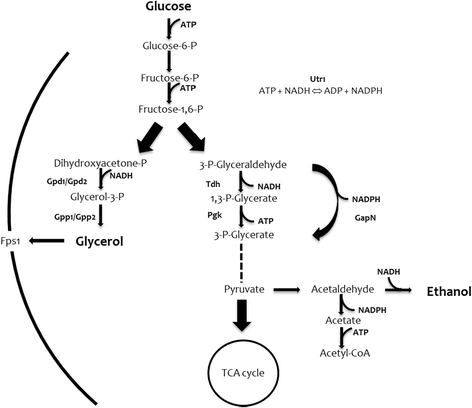

Supplement: Supplementary file 1 — Authors’ original file for figure 1 [file 13568_2014_86_MOESM1_ESM.gif]

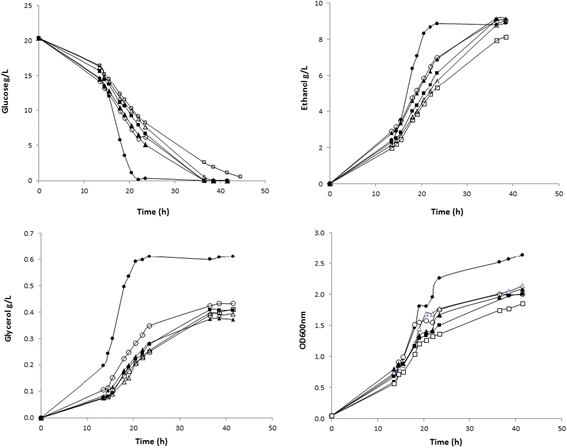

Supplement: Supplementary file 2 — Authors’ original file for figure 2 [file 13568_2014_86_MOESM2_ESM.gif]
